# An Evaluation of the Performance of Low-Cost Resin Printers in Orthodontics

**DOI:** 10.3390/biomimetics10040249

**Published:** 2025-04-18

**Authors:** Fırat Oğuz, Sabahattin Bor

**Affiliations:** Department of Orthodontics, Faculty of Dentistry, İnönü University, Malatya 44280, Türkiye; venaroshan@gmail.com

**Keywords:** orthodontic models, 3D printing, trueness, precision, digital dentistry

## Abstract

Background/Objectives: This study evaluated the trueness and precision of three low-cost 3D printers compared to a professional-grade printer in fabricating orthodontic models. Methods: Two upper dental models, one crowded and one non-crowded, were designed using Blenderfordental and Autolign. The models were printed with Anycubic M3 Premium, Anycubic Photon D2, Phrozen Sonic Mini 8K, and Ackuretta Sol at 45° and 90° using Elegoo orthodontic and Ackuretta Curo resins. A total of 384 models were produced: 256 crowded (128 at 90° and 128 at 45°) and 128 non-crowded (all at 45°). Chitubox Dental Slicer and ALPHA AI slicer were used for slicing. Post-processing involved cleaning with Ackuretta Cleani and curing in Ackuretta Curie. The models were scanned with Smartoptics Vinyl Open Air. Trueness was assessed using RMS deviation analysis in CloudCompare and linear measurements. Results: One-way ANOVA showed significant differences in trueness among the printers at 45° (*p* < 0.001) and 90° (*p* < 0.001). The Ackuretta Sol (LCD) exhibited the highest trueness, with the lowest mean RMS values at 45° (0.095 ± 0.008 mm) and 90° (0.115 ± 0.010 mm). The Anycubic M3 Premium (LCD) had the lowest trueness, with RMS values at 45° (0.136 ± 0.015 mm) and 90° (0.149 ± 0.012 mm). The 45° build angle resulted in significantly better trueness than 90° (*p* < 0.001). In linear measurements, deviations exceeding 0.25 mm were observed only in the R1 distance, except for the Ackuretta SOL, which remained below this threshold. Conclusions: The professional-grade printer demonstrated the best performance overall. Printing at a 45° build angle resulted in improved accuracy. Despite differences among devices, all printers produced results within clinically acceptable limits for orthodontic use.

## 1. Introduction

With recent developments in digital dentistry, the way has been paved for more innovative treatment planning services, such as the use of computer-aided design technology in orthodontic applications [1]. Three-dimensional (3D) printers constitute an important step in these innovations. Three-dimensional printer technology is an interdisciplinary technology that enables the three-dimensional production of any object [2]. In this technology, the object to be produced is first designed with a 3D software program and then sliced into layers and finally printed layer by layer using 3D printers [3]. This additive manufacturing is commonly referred to as three-dimensional (3D) printing technology [4].

In contrast to a subtractive manufacturing process, additive manufacturing can directly produce complex three-dimensional structures with increased manufacturing accuracy, a simplified manufacturing process, economic material and human resources usage, reduced manufacturing time, and enhanced production efficiency [5,6]. Layered manufacturing or 3D printing technology has been in development for a long time and has reached a level of maturity that allows its transformation into commercial applications in various industries, including automotive, aerospace, and biomedical engineering. In the field of dentistry, 3D printing technology has begun to be used in many areas, including restorative dentistry, prosthodontics, oral and maxillofacial surgery, and orthodontics [7].

The rapidly growing 3D printing technology, which is increasingly being used in various fields of dentistry, has also become an essential tool in orthodontics. This technology plays a significant role in the fabrication of orthodontic models, appliances, clear aligners, surgical splints, occlusal splints, and mini-screw insertion guides, enhancing precision and efficiency in treatment planning and execution [8,9,10].

In dentistry, 3D printing is generally utilized through vat polymerization techniques, which include Stereolithography (SLA), Digital Light Processing (DLP), and Liquid Crystal Display (LCD) methods [11], all of which dominate the layer-by-layer fabrication of dental objects through photopolymerization, offering superior accuracy and detail reproduction in various dental applications [12].

While SLA printing is more commonly used in scientific fields and laboratories, DLP and LCD printing are more common for home use. All three technologies rely on the photopolymerization of liquid resin [12]. Most of these resins consist of acrylate types that polymerize rapidly at room temperature.

In the SLA technique, which is the oldest method of 3D printing, the liquid resin is scanned and polymerized with a UV laser [13].

In the DLP technique, each layer is polymerized simultaneously using conventional light, which shortens the production time compared to the SLA technique [14]. For this reason, DLP-based 3D printing is widely used in dentistry, particularly in areas that require detailed work [15]. In this technique, projection light is used to polymerize materials and manufacture pre-designed models. A DMD (Digital Micromirror Device) with hundreds of thousands of tiny mirrors directs light. A DMD builds the optical model for DLP 3D printing inside the projector lens [16].

In the LCD technique, unlike DLP, the imaging system is a Liquid Crystal Display instead of a projector [12]. In LCD printing, an LCD screen is placed directly under the resin vat, where the direct contact of the screen with the vat prevents optical distortions. Printing also occurs layer by layer, and the resolution depends on the pixel size of the screen [17,18]. However, special attention must be given to the loss of light energy due to absorption by the screen, as approximately 90% of the light is absorbed by the LCD screen [19]. Additionally, because the screen is in direct contact with the bottom of the vat, controlling the oxygen level in the polymerization zone is not possible, which can lead to printing errors [12]. Although each of the three primary 3D printing techniques has its advantages and disadvantages, studies generally report that clinically acceptable and applicable models can be produced using 3D printers [20]. DLP and SLA technologies are generally known for their high accuracy and are therefore often preferred in dental applications that require fine detail. In contrast, LCD printers have gained popularity in recent years due to their lower cost and significant improvements in resolution and build quality.

The 3D printing of orofacial structures allows for extraoral treatment planning and the fabrication of therapeutic appliances on these models. Replicating biological structures provides significant advantages in both diagnosis and treatment planning, enabling detailed orthodontic evaluations outside the oral cavity. Furthermore, testing fabricated clear aligners, guides, or appliances on the printed models before intraoral application contributes to their safe and effective use.

In line with this, the use of DLP- and LCD-based 3D printers has become increasingly popular in dentistry. Among these techniques, LCD printing stands out as the most cost-effective option. However, in recent years, more affordable DLP 3D printer models have also emerged, making high-precision printing more accessible. One example is the Anycubic Photon D2, a DLP-based 3D printer that has the potential to provide accurate printouts while maintaining an accessible price point.

The aim of this study was to evaluate and compare the trueness and precision of orthodontic models (with and without crowding) produced by four different 3D printers with different printing technologies. The study hypothesized that there was a statistically significant difference in the trueness and precision of orthodontic models (with and without crowding) based on the 3D printing technologies and build angles used.

## 2. Materials and Methods

### 2.1. Model Design

**Figure 1:**
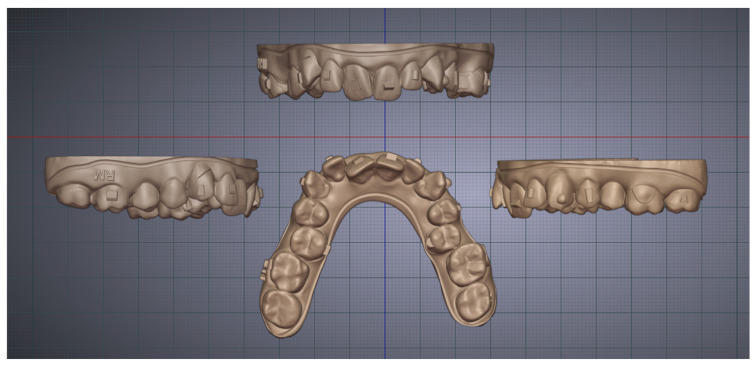
Crowded model in Blenderfordental.

**Figure 2:**
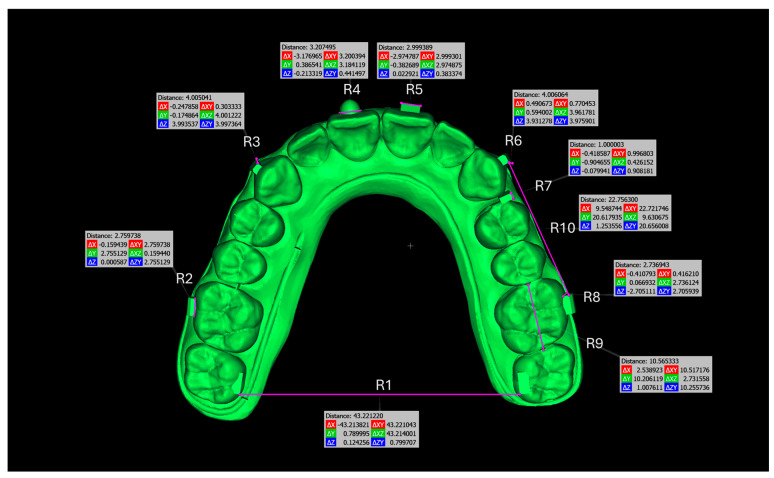
Non-crowded model with reference linear distances in Cloudcompare.

Two maxillary dental models were designed using Blenderfordental (B4D, version 1.1.24, 2024; Dubai, United Arab Emirates) and Autolign (Diorco; Seoul, Republic of Korea) (Figure 1 and Figure 2). The first model represented a crowded upper dental arch with attachments, enabling a detailed assessment of 3D printer performance. All models were printed using four different 3D printers (Table 1).

In contrast, the second model was a non-crowded upper dental model with attachments designed specifically to measure reference linear distances and assess the trueness of the printed models. This evaluation was conducted by comparing the measured reference linear distances on the printed models with those on the reference model. Manual measurements were digitally performed using CloudCompare (version 2.13.2 Kharkiv), focusing on specific reference linear distances to assess dimensional trueness (Figure 2).

### 2.2. Printing Material

All models were printed using Elegoo orthodontic model resin (Eleggo Inc., Shenzhen, China), except for those printed with the Ackuretta Sol (Ackuretta Technologies, Taipei, Taiwan), which utilized Ackuretta Curo model resin.

### 2.3. Printing Process

**Figure 3:**
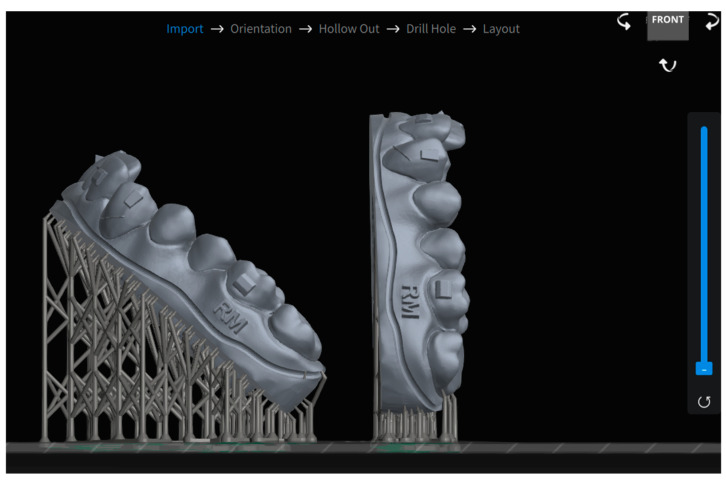
Comparative images of models oriented at 45° and 90° angles using Chitubox Dental.

For the crowded reference model, a total of 256 prints were produced: 128 oriented at 90 degrees and 128 inclined at 45 degrees, with both orientations printed on the same build plate (Figure 3). In contrast, for the non-crowded model, only 45-degree inclined prints were produced, resulting in a total of 128 models.

Chitubox Dental Slicer software v1.2.0 (Shenzhen, China) was used to prepare all 3D models for printing. Supports were added to prevent collapse during printing, and the slicing parameters, including a layer height of 100 microns, were adjusted according to each printer’s specifications. The supported models were then imported into ALPHA AI, the proprietary slicer program for the Ackuretta Sol printer.

### 2.4. Post-Processing

The printed models underwent a two-stage cleaning process using the Ackuretta Cleani system (Ackuretta Technologies, Taipei, Taiwan), with each stage involving a 5 min bath in 99% isopropyl alcohol. Following this, the models were cured for 10 min in the Ackuretta Curie (Ackuretta Technologies, Taipei, Taiwan).

### 2.5. Scanning

The Smartoptics Vinyl Open Air lab-type scanner (Smartoptics, Bochum, Germany) was used to digitize the printed models, with an accuracy of 6 µm (according to ISO 12836) [21].

### 2.6. Comparison and Evaluation

Digitally scanned models were compared to the reference models using B4D and CloudCompare (version 2.13.2 Kharkiv). Initially, both the reference and scanned models were imported into B4D and aligned using a landmark-based registration method. Following this, undesired parts of the models were trimmed according to a previously defined boundary (close to the aligner margin), ensuring that only the relevant regions were included in the comparison.

**Figure 4:**
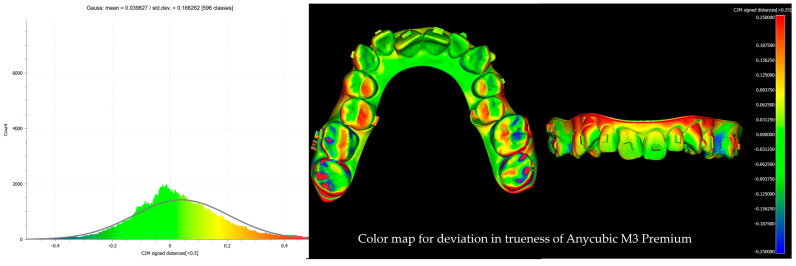
Color map with histogram for deviation in trueness of Anycubic M3 Premium. The scalar field shows ±0.25 mm deviations, where blue indicates inward deviations and red indicates outward deviations.

**Figure 5:**
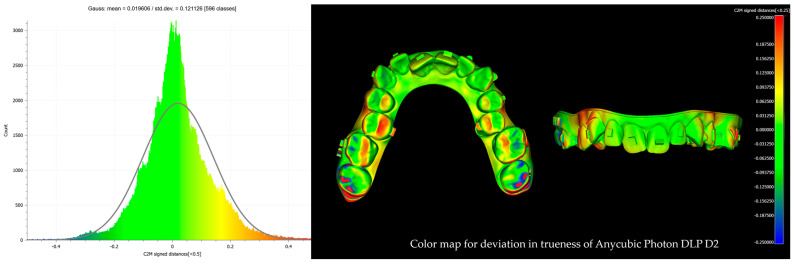
Color map with histogram for deviation in trueness of Anycubic Photon DLP D2. The scalar field shows ±0.25 mm deviations, where blue indicates inward deviations and red indicates outward deviations.

**Figure 6:**
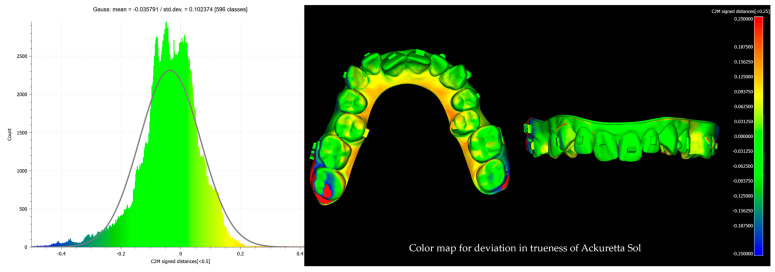
Color map with histogram for deviation in trueness of Ackuretta SOL. The scalar field shows ±0.25 mm deviations, where blue indicates inward deviations and red indicates outward deviations.

**Figure 7:**
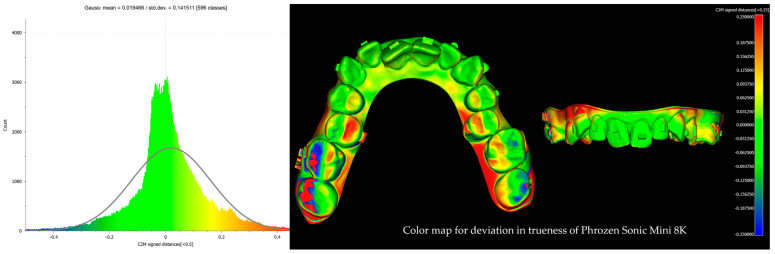
Color map with histogram for deviation in trueness of Phrozen Sonic Mini 8K. The scalar field shows ±0.25 mm deviations, where blue indicates inward deviations and red indicates outward deviations.

After alignment and trimming, the models were imported into CloudCompare, where precise surface alignment was achieved through fine registration using the Iterative Closest Point (ICP) algorithm. Subsequently, Root Mean Square (RMS) values were calculated to quantify surface deviations. This evaluation focused on two key metrics: trueness, which measures how accurately the printed models replicate the reference models, and precision, which assesses the consistency of repeated prints from the same printer by analyzing deviations across multiple print runs. Deviations were examined using RMS values and color maps with histograms (Figure 4, Figure 5, Figure 6 and Figure 7). A ±0.25 mm scalar field was applied to the color maps, where blue indicates inward deviations and red indicates outward deviations. To visualize the full distribution of deviations, a wider range of ±0.5 mm was used for the histograms.

### 2.7. Statistical Analysis

Descriptive statistics, including the mean and standard deviation (SD), were used. The Shapiro–Wilk test was performed to assess data distribution, while Levene’s test was used to evaluate homogeneity. Since four different printers were analyzed, one-way ANOVA was applied to determine whether there were significant differences between groups. For post hoc analysis, Tamhane’s T2 test was used to identify specific group differences. For linear distance measurements, the Kruskal–Wallis test was applied when normality assumptions were not met, and Dunn’s test was used for post hoc pairwise comparisons. To evaluate the effect of model orientation on print accuracy, a paired *t*-test was conducted to compare the accuracy between 45° and 90° orientations. All statistical analyses were performed using RStudio (2024.12.1 + 563), with a significance level set at *p* < 0.05.

The reliability of measurements in linear measurements was assessed using the Intraclass Correlation Coefficient (ICC) for both intra-operator and inter-operator reliability. The ICC value for intra-operator reliability was ICC: 0.92, which was the lowest among the parameters, while for inter-operator reliability, the lowest ICC value was ICC: 0.84.

## 3. Results

**Figure 8:**
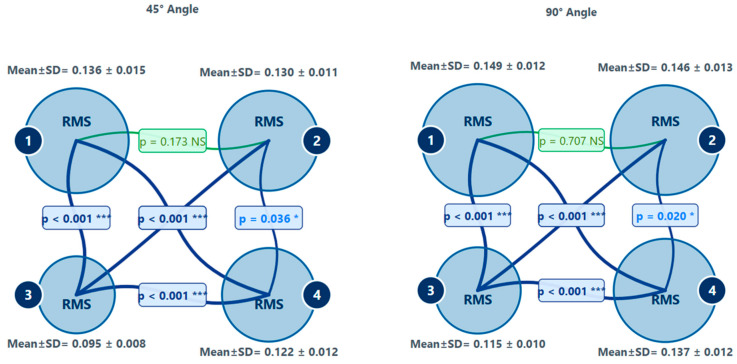
Pairwise comparison between printers at two build angles with respect to RMS values. Post hoc Tukey HSD test, *p* < 0.001 (***), *p* < 0.05 (*), *p* > 0.5 (NS: Not Significant), SD: standard deviation. 1: Anycubic M3 Premium, 2: Anycubic Photon D2, 3: Ackuretta SOL, 4: Phrozen Sonic Mini 8K, RMS: Root Mean Square.

One-way ANOVA, which assumes normal distribution and homogeneity of variance, revealed statistically significant differences among the four printers for RMS values at both 45° (F (3124) = 72.433; *p* < 0.001) and 90° (F (3124) = 56.318; *p* < 0.001). As the assumption was met, post hoc Tukey HSD comparisons were conducted to determine pairwise differences among the printers. The results indicated that at 45°, Printer 3 had significantly better trueness (lowest RMS, mean = 0.095 ± 0.008) compared to Printer 1 (mean difference = −0.041; *p* < 0.001), Printer 2 (mean difference = −0.035; *p* < 0.001), and Printer 4 (mean difference = −0.027; *p* < 0.001). Similarly, at 90°, Printer 3 showed significantly better trueness (mean = 0.115 ± 0.010) compared to Printer 1 (mean difference = −0.034; *p* < 0.001), Printer 2 (mean difference = −0.031; *p* < 0.001), and Printer 4 (mean difference = −0.023; *p* < 0.001). Printer 1 consistently demonstrated the poorest trueness at both angles (45° mean = 0.136 ± 0.015; 90° mean = 0.149 ± 0.012). Printers 2 and 4 had intermediate trueness values, significantly different from both Printers 1 and 3 (all *p* < 0.05) (Figure 8).

When evaluating the effect of the build angle on print trueness, lower RMS values were found to be significantly better at 45° compared to 90° for all printers (*p* < 0.001) (Table 2). These findings indicate that the 45° build angle provided greater trueness across all printers.

Statistical analyses were performed on the data obtained through linear measurements to determine whether significant differences existed among different printer groups. ANOVA and Kruskal–Wallis tests were applied based on the distribution characteristics of the data.

To evaluate the distribution of the data, the Shapiro–Wilk test was conducted, revealing that some variables followed a normal distribution while others did not. Levene’s test was used to assess the homogeneity of variances, indicating that R1 had homogeneous variances, whereas R2 and R10 did not.

For variables that met the normality assumption, a one-way ANOVA test was applied to determine whether significant differences existed among the groups. The results showed statistically significant differences for R1, R2, and R10 (*p* < 0.05). Post hoc tests were conducted to determine which groups differed significantly from one another. Tukey HSD was applied for R1 due to homogeneous variances, while Tamhane’s T2 test was used for R2 and R10 due to non-homogeneous variances. The results indicated significant differences among all groups in R1 (*p* < 0.05), whereas in R2, only groups 3 and 4 differed (*p* = 0.022), and in R10, significant differences were observed in comparisons of 1–3 (*p* < 0.001), 2–3 (*p* < 0.001), 2–4 (*p* = 0.002), and 3–4 (*p* = 0.002) (Table 3).

For variables that did not meet normality assumptions, the Kruskal–Wallis test was performed. This test revealed significant differences for R3 (*p* < 0.001), R6 (*p* = 0.036), and R9 (*p* < 0.001), while R4 (*p* = 0.108), R5 (*p* = 0.849), R7 (*p* = 0.594), and R8 (*p* = 0.088) did not show significant differences (*p* > 0.05). Post hoc pairwise comparisons showed that in R3, Printer 1 significantly differed from Printers 2 and 3 (*p* < 0.05), while in R6, Printer 1 exhibited a significant difference from Printer 3 (*p* = 0.041). For R9, Printers 3 and 4 significantly differed from Printers 1 and 2 (*p* < 0.001), but no significant difference was found between Printers 3 and 4 (*p* = 1.000) (Table 3).

**Figure 9:**
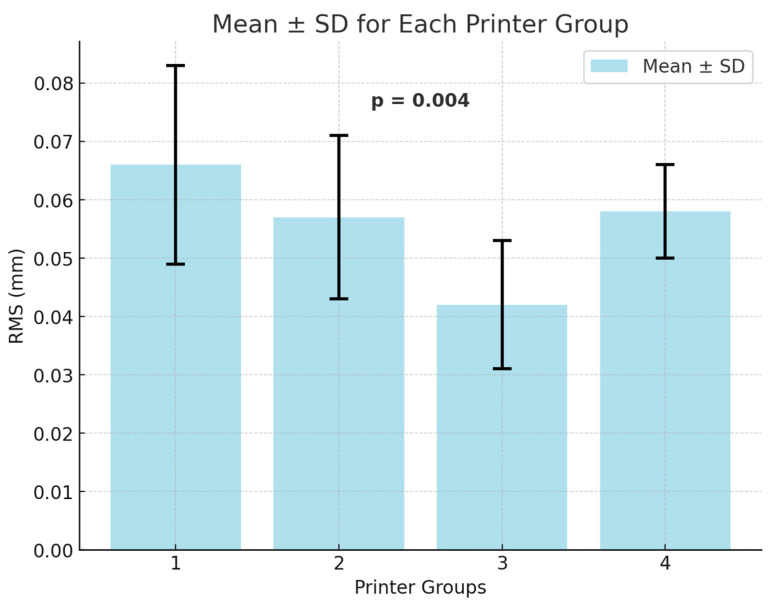
Precision evaluation across all printer groups by superimposing repeatedly printed models. 1: Anycubic M3 Premium, 2: Anycubic Photon D2, 3: Ackuretta SOL, 4: Phrozen Sonic Mini 8K. RMS: Root Mean Square, SD: standard deviation, mm: millimeter.

A one-way ANOVA test was conducted to compare the precision of different printers based on RMS values (Figure 9), revealing a statistically significant difference among the printers (*p* = 0.004). To determine which specific printers differed, a post hoc Tukey HSD test was performed. The results showed that the significant difference was between Printer 1 and Printer 3 (*p* = 0.002), while the other pairwise comparisons were not statistically significant.

## 4. Discussion

Three-dimensional printing technology is widely used in orthodontics for fabricating models, appliances, clear aligners, surgical and occlusal splints, and mini-screw guides, enhancing precision and efficiency in treatment planning [7,8,9,10]. The growing demand for clear aligner therapy has driven advancements in 3D printing, enabling precise aligner fabrication for accurate tooth movement and improved outcomes [22]. Additionally, 3D printing allows for fully customized orthodontic appliances, enhancing fit, comfort, and clinical effectiveness while reducing chairside adjustments and improving patient compliance [23].

Layer thickness and model orientation angles can influence the 3D printing process [24]. Ko et al. [25] reported that although most DLP-printed models were within clinically acceptable limits, the smallest layer height (20 µm) resulted in the least accurate prints. Similarly, Favero et al. [26] found that, in SLA printing, models printed at 25 µm exhibited the greatest deviations, whereas those printed at 100 µm were the most accurate.

However, another study reported that, in horizontal printing, 25 µm yielded better results with an SLA printer, while 175 µm was more accurate with a DLP printer. Since our study did not involve SLA printers and aimed to compare a DLP printer with several LCD printers, we adopted a 100 µm layer height. This choice also aligns with prior research showing no significant difference in print accuracy between 50 and 100 µm in LCD technology [25].

Model anatomy may also impact print quality [11,27]. One study suggested accuracy differences between aligned and crowded teeth models [27]. Therefore, two models were printed: one with crowding and another without. The crowded model, featuring attachments, was assessed using RMS values from a superimposition method.

Different 3D printing techniques can impact model accuracy, leading to comparative studies [24]. ElShebiny et al. [24] found that while both DLP and SLA printers produce clinically acceptable orthodontic models, DLP offers greater precision. Unlike the study by ElShebiny et al. [24], this study evaluated three LCD and one DLP printer, which are more commonly used today instead of SLA. In addition, both crowded and non-crowded models were compared, and manual linear measurements were performed alongside RMS analysis.

Grassia et al. [11] compared SLA, DLP, and LCD technologies, reporting that SLA had the highest accuracy, DLP the highest precision, and LCD the lowest accuracy. Their study printed all models at a single build angle without support structures and evaluated accuracy solely through RMS analysis. In contrast, the present study utilized both 45° and 90° build angles with support structures and incorporated manual linear measurements alongside RMS analysis [11].

LCD and DLP technologies are widely used in dentistry, particularly for orthodontic modeling, appliance fabrication, and clear aligner production, due to their high resolution and speed [28]. However, to the best of our knowledge, previous studies did not include entry-level DLP printers, and the LCD printers evaluated in those studies lacked the high screen resolution featured in the printers used in our study [29,30].

DLP printers are widely known for their longer screen lifespans compared to LCD printers. However, in our study, the Ackuretta SOL, which is an LCD printer, was reported by its manufacturer to have a screen lifespan of up to 10,000 h. This is significantly longer than the typical lifespan of most other LCD printers, which is generally limited to around 2000 h.

In our study, the Ackuretta SOL (LCD, Printer 3) demonstrated the highest trueness and precision, with the lowest RMS error values and minimal variability at both printing angles. The Phrozen Sonic Mini 8K (LCD, Printer 4) and the Anycubic Photon D2 (DLP, Printer 2) showed higher RMS values compared to the Ackuretta SOL, indicating comparatively lower trueness and precision. The Anycubic M3 Premium (LCD, Printer 1) exhibited the highest error rates and variability among all printers, reflecting the lowest performance in both trueness and precision.

To evaluate how accurately the planned tooth movements for clear aligners were transferred to the printed models, nine reference linear distances were assessed. Among these references, R1 was measured from the corners of the rectangles placed on the palatal surfaces of the second molars. Although the average RMS values in the crowded model were below 0.25 mm, in linear measurements on the non-crowded model, a deviation exceeding 0.5 mm was observed in the R1 distance of the Anycubic M3 Premium. This deviation is likely attributable to expansion occurring in the posterior region during printing. Notably, only the Ackuretta SOL achieved an R1 deviation below 0.25 mm, while the other printers exceeded this threshold. However, for the remaining reference linear distances, all printers exhibited deviations below 0.25 mm (Table 3). While the Ackuretta SOL generally demonstrated the best performance based on surface superimposition, this superiority was not consistent across all linear measurements. These discrepancies may be due to the limited number and distribution of the linear measurement points. Color deviation maps further support this interpretation, revealing greater deviations in certain localized areas and minimal discrepancies in others.

The deviation observed in the Anycubic M3 Premium (8K) printer is thought to be related to the increased size of the build plate. In terms of precision, a significant difference was observed only between the Ackuretta SOL and the Anycubic M3 Premium.

Lohfeld et al. [31] compared the accuracy of dental models printed at different angles (0°, 70°, and 90°) and reported that the highest accuracy was achieved at 70°. While 0° printing resulted in clinically unacceptable deviations in certain measurements (e.g., canine height), the 70° angle provided better accuracy than both 0° and 90°. This was attributed to more favorable layer distribution and support placement, minimizing distortion along the *Z*-axis and critical surfaces.

The present study found that printing at a 45° angle improved overall print accuracy. This improvement may be attributed to the increased number of layers along the *Z*-axis, which could lead to the accumulation of errors, prolonged printing time resulting in greater exposure to gravity, and the potential influence of *Z*-axis movement accuracy on print quality. Another possible factor is the inability to calibrate the resin optimally in terms of exposure time.

In models printed at a 90° angle, support marks may have contributed to increased deviation errors during superimposition. This can be considered another possible explanation for the higher deviations observed at this angle, as the support structures often overlapped with the superimposition areas, potentially increasing the deviation and affecting the accuracy of the measurements. In contrast, at a 45° angle, the support marks remained outside the superimposition zones and did not appear to influence the deviation results.

Many studies consider an error margin of 0.2–0.5 mm acceptable for clinical accuracy [32,33]. However, for clear aligner fabrication, stricter tolerances are required. Since each aligner enables 0.25–0.30 mm of tooth movement per stage [34], the dimensional discrepancy between the printed model and its original should be less than 0.25–0.30 mm to ensure accurate force application. Thus, for clear aligner production, a 0.25 mm average error is deemed clinically acceptable [35]. Lohfeld et al. [31] also defined ±0.25 mm as an acceptable margin for intra-tooth measurements and ±0.5 mm for inter-tooth (cross-arch) measurements.

In our study, all printers, regardless of budget level, maintained clinically acceptable accuracy, with error rates below 0.25 mm. Similarly, Nulty [29] compared 12 different 3D printers across various price ranges, using 100-micron test blocks. While significant differences were observed in average errors, he concluded that all printers performed acceptably. Unlike our study, Nulty used cube-shaped prints rather than dental models to assess accuracy and precision. In contrast, we employed two distinct maxillary models to better simulate dental tissues, providing a more realistic evaluation of 3D printing accuracy for orthodontic applications.

Lo Giudice et al. [30] and Venezia et al. [27] both compared the accuracy of four different 3D printing technologies, including a DLP printer, an entry-level LCD printer, and two SLA printers. In the studies by Lo Giudice et al. [30] and Venezia et al. [27], the models were printed at a single angle without support structures, and only RMS analysis was performed. In contrast, the present study printed models at 45° and 90° angles with support structures and conducted manual linear measurements in addition to RMS analysis. They found that all tested printers produced orthodontic models with accuracy and precision errors below 0.25 mm, meeting clinical acceptability standards.

However, previous studies have highlighted that entry-level LCD printers exhibit accuracy and precision errors approaching the clinical threshold, indicating that extreme caution is warranted when using such devices for clear aligner fabrication [11,27,30]. In contrast, the entry-level LCD and DLP printers used in our study demonstrated improved performance. We attribute this improvement primarily to the higher screen resolution (8K) and enhanced technical specifications of the printers. Nevertheless, it should also be noted that differences in the type of resin and the geometry of the printed models may have influenced the results.

In the present study, Root Mean Square (RMS) analysis was used to evaluate printing accuracy. While the RMS method measures the overall amount of error across the entire surface, it does not provide a detailed analysis of localized errors, which can be considered a limitation of our study. We addressed this issue by measuring linear distances. We used the Curo model resin for the Ackuretta Sol because this resin had already been calibrated for this printer system. For future studies, we recommend comparing printers using the same resin. Another limitation of our study is the potential influence of support structures on deviation errors.

This study focused on assessing model accuracy from an orthodontic perspective. In this context, two printing angles were selected: 90°, which allows the simultaneous production of multiple models in a single print, and 45°, which is suitable for evaluating accuracy in relation to average model height. A 0° printing angle was not included, as it was considered impractical for in-house aligner production with the printers used, particularly those with relatively small build plates. Therefore, it was deemed unsuitable for the scope of this study. However, while the exclusion of the 0° angle is acknowledged as a limitation, future studies exploring this angle may prove valuable, especially for printers with larger build plates or mid-range devices such as the Anycubic M3 Premium.

## 5. Conclusions

All printers provided clinically acceptable results for orthodontic use. However, Ackuretta SOL showed the highest accuracy and precision, while the Anycubic M3 Premium (LCD) had the highest error rate and produced less consistent outcomes. Although both build angles yielded acceptable results, the 45° angle was more accurate. Furthermore, in linear measurements, only the Anycubic M3 Premium exceeded a 0.5 mm deviation at the R1 reference distance.

## Figures and Tables

**Table 1:** The printers used in the study and their specifications.

Feature	Anycubic Photon M3 Premium (Printer 1)	Anycubic Photon DLP D2 (Printer 2)	Ackuretta SOL (Printer 3)	Phrozen Sonic Mini 8K (Printer 4)
3D Printing Technology	Monochrome LCD	DLP (Digital Light Processing)	Monochrome LCD	Monochrome LCD
Screen/Projector	7680 × 4320 pixels (8K)	2560 × 1440 pixels (2K) DLP Projector	Unspecified	7500 × 3240 pixels (8K)
XY Resolution	28.5 µm	51 µm	49 µm	22 µm
Layer Thickness (Adjustable)	50–150 µm	50–150 µm	50–150 µm	22–150 µm
Price	USD 650–400	USD 680–400	USD 9750	USD 509.99
Screen Lifespan	2000 h	20000 h	10000 h	2000 h

**Table 2:** Comparison of printers based on two build angles.

Printer	(45° vs. 90°)	*t*-Value	*p*-Value
**1**	45° vs. 90°	−4.044	<0.001 ***
**2**	45° vs. 90°	−4.713	<0.001 ***
**3**	45° vs. 90°	−8.912	<0.001 ***
**4**	45° vs. 90°	−5.905	<0.001 ***

Paired *t*-test, *p* < 0.001 (***), 1: Anycubic M3 Premium, 2: Anycubic Photon D2, 3: Ackuretta Sol, 4: Phrozen Sonic Mini 8K.

**Table 3:** Comparison of printers’ trueness based on linear measurements.

Variable	Test	*p*-Value	Post Hoc	Printer 1	Printer 2	Printer 3	Printer 4	Group Differences
R1	ANOVA	<0.05	Tukey HSD	0.533 ± 0.067	0.294 ± 0.049	0.204 ± 0.043	0.318 ± 0.066	All groups significantly different
R2	ANOVA	<0.05	Tamhane T2	0.035 ± 0.039	0.021 ± 0.020	0.013 ± 0.024	0.043 ± 0.041	Only 3–4 different
R3	Kruskal–Wallis	<0.001	Dunn’s	0.04 (−0.04–0.12)	0.02 (−0.04–0.05)	0.01 (−0.04–0.03)	0.02 (−0.03–0.06)	1–2, 1–3 different
R4	Kruskal–Wallis	0.108	None	0.04 (−0.05–0.10)	0.03 (−0.05–0.05)	0.02 (0.00–0.07)	0.02 (0.01–0.04)	No significant differences
R5	Kruskal–Wallis	0.849	None	0.02 (−0.04–0.05)	0.02 (−0.04–0.05)	0.02 (−0.02–0.04)	0.02 (−0.04–0.04)	No significant differences
R6	Kruskal–Wallis	0.036	Dunn’s	0.02 (−0.03–0.05)	0.01 (−0.03–0.03)	0.01 (0.00–0.02)	0.01 (−0.03–0.04)	Only 1–3 different
R7	Kruskal–Wallis	0.594	None	0.01 (−0.03–0.02)	0.01 (−0.01–0.02)	0.01 (−0.01–0.03)	0.01 (−0.01–0.03)	No significant differences
R8	Kruskal–Wallis	0.088	None	0.03 (−0.04–0.07)	0.03 (−0.04–0.05)	0.01 (−0.05–0.03)	0.02 (−0.04–0.05)	No significant differences
R9	Kruskal–Wallis	<0.001	Dunn’s	0.11 (−0.11–0.18)	0.12 (0.07–0.16)	0.04 (0.01–0.07)	0.04 (0.01–0.11)	1–2, 1–3, 1–4, 2–4 different
R10	ANOVA	<0.05	Tamhane T2	0.151 ± 0.038	0.167 ± 0.032	0.087 ± 0.042	0.127 ± 0.022	1–3, 2–3, 3–4 different

Note: Printers with the same color are NOT significantly different from each other (*p* > 0.05). Printers with different colors are significantly different from each other (*p* < 0.05). For parametric tests (ANOVA), values are presented as mean ± SD. For non-parametric tests (Kruskal–Wallis), values are presented as median (Min-Max). 1: Anycubic M3 Premium, 2: Anycubic Photon D2, 3: Ackuretta SOL, 4: Phrozen Sonic Mini 8K.

## Data Availability

The original contributions presented in the study are included in the article; further inquiries can be directed to the corresponding author.
